# Identification and experimental validation of programmed cell death- and mitochondria-associated biomarkers in osteoporosis and immune microenvironment

**DOI:** 10.3389/fgene.2024.1439171

**Published:** 2024-07-26

**Authors:** Xiu Yang, Zheng-Chao Zhang, Yun-Nan Lu, Han-Lin Chen, Hong-Shen Wang, Tao Lin, Qing-Quan Chen, Jin-Shui Chen, Wu-Bing He

**Affiliations:** ^1^ Shengli Clinical Medical College of Fujian Medical University, Fuzhou, China; ^2^ Fuzong Clinical Medical College of Fujian Medical University, Fuzhou, China; ^3^ Department of Emergency Trauma Surgery, Fujian Provincial Hospital, Fuzhou, China; ^4^ Fujian Trauma Medicine Center, Fuzhou, China; ^5^ Fujian Key Laboratory of Emergency Medicine, Fuzhou, China; ^6^ Department of Paediatric Orthopaedics, Fuzhou Second Hospital, The Third Clinical Medicine College of Fujian Medical University, Fuzhou, China

**Keywords:** osteoporosis, programmed cell death, mitochondria, nomogram, machine learning

## Abstract

**Background:** Prior research has demonstrated that programmed cell death (PCD) and mitochondria assume pivotal roles in controlling cellular metabolism and maintaining bone cell equilibrium. Nonetheless, the comprehensive elucidation of their mode of operation in osteoporosis (OP) warrants further investigation. Therefore, this study aimed at analyzing the role of genes associated with PCD (PCD-RGs) and mitochondria (mortality factor-related genes; MRGs) in OP.

**Methods:** Differentially expressed genes (DEGs) were identified by subjecting the GSE56815 dataset obtained from the Gene Expression Omnibus database to differential expression analysis and comparing OP patients with healthy individuals. The genes of interest were ascertained through the intersection of DEGs, MRGs, and PCD-RGs; these genes were filtered using machine learning methodologies to discover potential biomarkers. The prospective biomarkers displaying uniform patterns and statistically meaningful variances were identified by evaluating their levels in the GSE56815 dataset and conducting quantitative real-time polymerase chain reaction-based assessments. Moreover, the functional mechanisms of these biomarkers were further delineated by constructing a nomogram, which conducted gene set enrichment analysis, explored immune infiltration, generated regulatory networks, predicted drug responses, and performed molecular docking analyses.

**Results:** Eighteen candidate genes were documented contingent upon the intersection between 2,354 DEGs, 1,136 MRGs, and 1,548 PCD-RGs. The biomarkers *DAP3*, *BIK*, and *ACAA2* were upregulated in OP and were linked to oxidative phosphorylation. Furthermore, the predictive ability of the nomogram designed based on the OP biomarkers exhibited a certain degree of accuracy. Correlation analysis revealed a strong positive correlation between CD56dim natural killer cells and *ACAA2* and a significant negative correlation between central memory CD4^+^ T cells and *DAP3*. *DAP3*, *BIK*, and *ACAA2* were regulated by multiple factors; specifically, SETDB1 and ZNF281 modulated *ACAA2* and *DAP3*, whereas TP63 and TFAP2C governed *DAP3* and *BIK*. Additionally, a stable binding force was observed between the drugs (estradiol, valproic acid, and CGP52608) and the biomarkers.

**Conclusion:** This investigation evidenced that the biomarkers *DAP3*, *BIK*, and *ACAA2* are associated with PCD and mitochondria in OP, potentially facilitate the diagnosis of OP in clinical settings.

## 1 Introduction

Osteoporosis (OP) is a persistent condition characterized by reduced bone density, degradation of bone composition, and fractures resulting from brittleness (Walker and Shane, 2023). This condition can lead to a diminished quality of life, premature mortality, disability, and an elevated economic burden (Reid and Billington, 2022; Ensrud and Crandall, 2024). The current clinical management of OP predominantly involves the administration of medications such as bisphosphonates, estrogen therapies, and calcitonin (van der Burgh et al., 2021). Nevertheless, adverse reactions associated with these medications impede their ability to effectively remediate OP. The etiology of OP is complex, with disturbances in bone metabolism increasing the likelihood of developing the disease. With advancements in research on osteoimmunology and the molecular mechanisms related to OP, there is growing acknowledgment of the significance of molecular and osteoimmune interventions in treating OP (Adejuyigbe et al., 2023). Consequently, this exerts an urgent requirement to develop innovative therapies for the treatment of OP.

Programmed cell death (PCD) is an active process through which cells die in response to specific signals or stimuli to maintain the balance of the internal environment. PCD is involved in various biochemical and morphological characteristics (Li Z. et al., 2023). The different types of PCD include apoptosis, necrosis, autophagy, lysosome-dependent cell death, ferroptosis, necroptosis, pyroptosis, and immunogenic cell death (Bedoui et al., 2020; Awuti et al., 2022). Under normal physiological conditions, PCD occurs in harmony to sustain cell stability. However, in conditions such as OP, imbalances in PCD types can arise and interact with each other, thereby affecting the onset and progression of OP. Efficiently controlled PCD plays a crucial role in maintaining osteoblast (OB) and bone metabolism stability, whereas excessive PCD can result in OB impairment, enhanced conversion of bone marrow mesenchymal stem cells (BMSCs) into adipocytes, and the accumulation of harmful substances, ultimately contributing to OP development (Daponte et al., 2024). Regulation of bone metabolism is significantly influenced by apoptosis, autophagy, ferroptosis, pyroptosis, and necrosis, all of which play crucial roles in bone cell activity (Zhang et al., 2023). Despite advancements in identifying essential molecules and signaling pathways involved in PCD, our understanding of how these pathways control the onset and progression of OP remains limited.

Mitochondria assume a crucial role in energy production, iron balance, and the synthesis of different compounds. Studies have shown that mitochondria are vital for regulating the equilibrium between osteoclast and OB functions, thereby affecting bone health (Wang et al., 2021). Furthermore, mitochondria govern cell growth, specialization, energy generation, signaling, and cell death. Any malfunction in mitochondrial function can lead to conditions such as OP and reduced OB activity, which are common in orthopedic ailments. Senile OP is linked to abnormal mitochondrial function, which hinders bone formation, increases osteoclast activity, and accelerates bone deterioration (Zheng et al., 2020). Mitochondria also participate in cell death, and irregular mitochondrial operations activate diverse forms of PCD (Flowers et al., 2023). The buildup of faulty mitochondria leads to the excessive production of reactive oxygen species (ROS), hastening cell death (Wang et al., 2012). Mitochondrial glycolysis and oxidative phosphorylation pathways, in conjunction with ROS generation, are essential for meeting the energy needs of bone-forming cell proliferation and differentiation (Qin et al., 2019). The intricate interplay between mitochondrial function and programmed cell death (PCD) plays a pivotal role in the pathogenesis of osteoporosis (OP). However, there is a dearth of bioinformatics research dedicated to elucidating and validating genes that are co-expressed with PCD and mitochondria during the progression of OP.

Therefore, this study aimed to elucidate and validate genes that exhibit co-expression with PCD and mitochondria during the progression of OP. Specifically, we employed a two-step approach: ① Identification and validation of markers using data from the Gene Expression Omnibus (GEO) database combined with quantitative real-time polymerase chain reaction (qRT-PCR) analysis on serum samples obtained from clinical subjects; ② Exploration of underlying mechanisms and prognostic potential through enrichment analyses, immune infiltration assessments, establishment of regulatory networks, and prediction of potential therapeutic drugs. In order to address the future clinical demands of osteoporosis treatment, it is imperative to enhance targeted regulation of mitochondrial function and programmed cell death, thereby fostering fundamental research and facilitating drug development in bone metabolism.

## 2 Materials and methods

### 2.1 Data extraction

Data pertaining to OP was acquired from the GEO database (http://www.ncbi.nlm.nih.gov/geo/). The GSE56815 dataset (platform: GPL96) served as the training set and comprised 40 blood samples (circulating monocytes), each derived from healthy individuals with high hip bone mineral density (BMD) and OP patients with low BMD. Each group of participants included 20 premenopausal and 20 postmenopausal women. A total of 1,548 PCD-related genes (PCD-RGs) and 1,136 genes associated with mitochondria (mortality factor-related genes; MRGs) were procured from extant literature (Supplementary Table S1) (Qin et al., 2023) and the MitoCarta 3.0 database (https://www.broadinstitute.org/mitocarta/mitocarta30-inventory-mammalian-mitochondrial-proteins-and-pathways), respectively.

### 2.2 Differential expression analysis

To identify differentially expressed genes (DEGs), the OP and control specimens within the GSE56815 dataset were subjected to differential expression analysis-based comparison using the limma package (v 3.57.11; significance threshold of *p* < 0.05) (Ritchie et al., 2015). Subsequently, the DEGs were visualized by generating volcano plots and heat maps using the ggplot2 (v. 3.4.2) (Gustavsson et al., 2022) and the ComplexHeatmap packages (v 2.17.0) (Gu et al., 2016), respectively.

### 2.3 Recognition and functional analysis of candidate genes

To distinguish the DEGs related to both PCD and mitochondria, the DEGs were overlapped with the MRGs and PCD-RGs using the ggVennDiagram package (v 1.2.3) (Gao et al., 2021). The resulting genes were denoted as potential candidate biomarker genes. Next, the aforementioned genes were annotated via Gene Ontology (GO) and pathway analyses using the clusterProfiler package (v 4.9.4) (Wu et al., 2021) with an adjusted *p*-value cutoff of <0.05. GO analysis encompassed biological processes (BPs), cellular components (CCs), and molecular functions (MFs). This was followed by an enrichment analysis using the Kyoto Encyclopedia of Genes and Genomes (KEGG) pathways. Additionally, the potential interactions between the selected genes were investigated by formulating a protein-protein interaction (PPI) network for the candidate genes using the STRING online database (https://string-db.org/) with an interaction score threshold of 0.4. The generated PPI network was displayed using Cytoscape version 3.7.1 (Shannon et al., 2003).

### 2.4 Biomarker identification and validation

The LASSO regression analysis was employed in conjunction with support vector machine recursive feature elimination (SVM-RFE) algorithms to define specific candidate genes; the LASSO regression analysis and the SVM-RFE algorithm were performed using the glmnet package (v 4.1-8) (Sasikumar et al., 2022) with binomial family parameters and the e1071 package (v 1.7-13) (Yang et al., 2022), respectively. Candidate biomarkers were identified by merging the genes discerned using the aforementioned methods. Subsequently, the expression of the candidate biomarkers was analyzed using the GSE56815 dataset, and their validity was confirmed using quantitative real-time polymerase chain reaction (qRT-PCR).

Blood samples belonging to 10 patients diagnosed with OP and 10 individuals lacking the condition were obtained from the Biobank of the 900th Hospital of the Joint Logistics Support Force and subjected to qRT-PCR analysis using the 2xUniversal Blue SYBR Green qPCR Master Mix (Servicebio, Wuhan, China). This study was approved by the Biomedical Ethics Committee of the 900th Hospital of the Joint Logistics Support Force (Ethics Review No. 2023-72), and all study participants provided informed consent. RNA was extracted from all 20 samples using TRIzol (Ambion, Austin, TX, United States) per manufacturer guidelines And subsequently converted into cDNA using the SureScript First Strand cDNA Synthesis Kit (Servicebio). The PCR primer sequences have been listed in Supplementary Table S2, with GAPDH serving as the internal reference gene. The expression of the candidate biomarkers was determined using the 2^−ΔΔCT^ method (Livak and Schmittgen, 2001). Biomarkers that displayed consistent trends in both the dataset and qRT-PCR results, as well as considerable differences between the OP and control groups, were identified and documented as potential biomarkers. The mRNALocator database (http://bio-bigdata.cn/mRNALocater/) was used to predict the subcellular positioning of these biomarkers, and the RCircos tool (v. 1.2.0) (Zhang et al., 2013) was used to examine their chromosomal dispersal.

### 2.5 Engineering and evaluation of nomogram

The rms software (version 6.7-1) was applied to biomarkers reported in a prior study (Xu et al., 2023) to construct a nomogram using the GSE56815 dataset and evaluate the combined potency of the candidate biomarkers to prognosticate OP. The predictive accuracy of the nomogram was evaluated using calibration, decision, and ROC curves.

### 2.6 Gene set enrichment analysis (GSEA)

Two background gene sets—c2.cp.kegg.v7.5.1. entrez.gmt for the KEGG signaling pathway and c5.go.bp.v2023.2—were initially considered to explore the roles and pathways linked to the biomarkers in OP progression. Hs.symbols.gmt was used for GO functional enrichment from the Molecular Signatures Database (https://www.gsea-msigdb.org/gsea/msigdb). The correlation coefficients between biomarkers and other genes within the GSE56815 dataset were determined, and subsequently, the genes were organized according to their correlation coefficients to establish a gene set for enrichment analysis. The identified biomarkers were then subjected to GSEA using the clusterProfiler package.

### 2.7 Immune infiltration analysis

To assess variations in immune status among OP patients, 28 immune cell infiltration scores between OP and control groups from the GSE56815 dataset were initially calculated using the ssGSEA algorithm of the GSVA package (v. 1.49.8) (Hänzelmann et al., 2013). The Wilcoxon test was used to compare discrepancies in the immune infiltration levels of these 28 immune cells between the OP and control groups. Subsequently, Spearman’s correlation analysis was performed to investigate the relationship between the biomarkers and diverse immune cells.

### 2.8 Construction of regulatory networks

To establish regulatory networks, the NetworkAnalyst database (https://www.networkanalyst.ca/) was used to predict the transcription factors (TFs) involved in the regulation of the recognized biomarkers; the predicted TFs were then intersected with the DEGs to identify the crucial TFs. Moreover, we utilized both the miRWalk (http://mirwalk.umm.uni-heidelberg.de/) and microT (https://dianalab.e-ce.uth.gr/microt_webserver/) databases to predict biomarker-targeting microRNAs (miRNAs). Subsequently, the results from these two databases were combined to determine the key miRNAs. Finally, the Cytoscape software was used to fashion networks representing mRNA-TF and mRNA-miRNA interactions.

### 2.9 Drug prediction and molecular docking analysis

The potential therapeutic capabilities of the candidate biomarkers were examined using the Comparative Toxicogenomics Database (http://ctdbase.org/) to identify prospective drugs targeting biomarkers with a screening criteria of reference count ≥1. Crystal structures of receptor proteins for the recognized biomarkers were obtained from UniProt (https://www.uniprot.org/) and the Protein Data Bank (PDB) database (https://www.rcsb.org/) in the PDB format. The 3D structures of the targeted drugs were downloaded as ligands from PubChem (https://pubchem.ncbi.nlm.nih.gov/), energy minimized using the OpenBabel software (v. 2.4.1) (Mehta et al., 2014) and saved in the mol2 format. PyMOL software (v 3.3.0) (Seeliger and de Groot, 2010) was used to dehydrate, de-ligandize, and hydrogenate the receptor proteins prior to saving them. The processed receptor proteins and target drug structures were then imported into the AutoDock Tools software (v 1.5.7) (Morris et al., 2009) and converted into the pdbqt format. Finally, molecular docking was performed using the AutoDock Vina software (v 1.2.2) (Trott and Olson, 2010), and the results were visualized using PyMOL software.

### 2.10 Statistical analysis

R software (4.2.2 version) was used for data processing and analysis. The Wilcoxon test was used to compare variances among diverse groups. A significance level of *p* < 0.05 was deemed statistically noteworthy.

## 3 Results

### 3.1 Eighteen candidate genes were associated with mitochondrial-related signaling pathways

**FIGURE 1:**
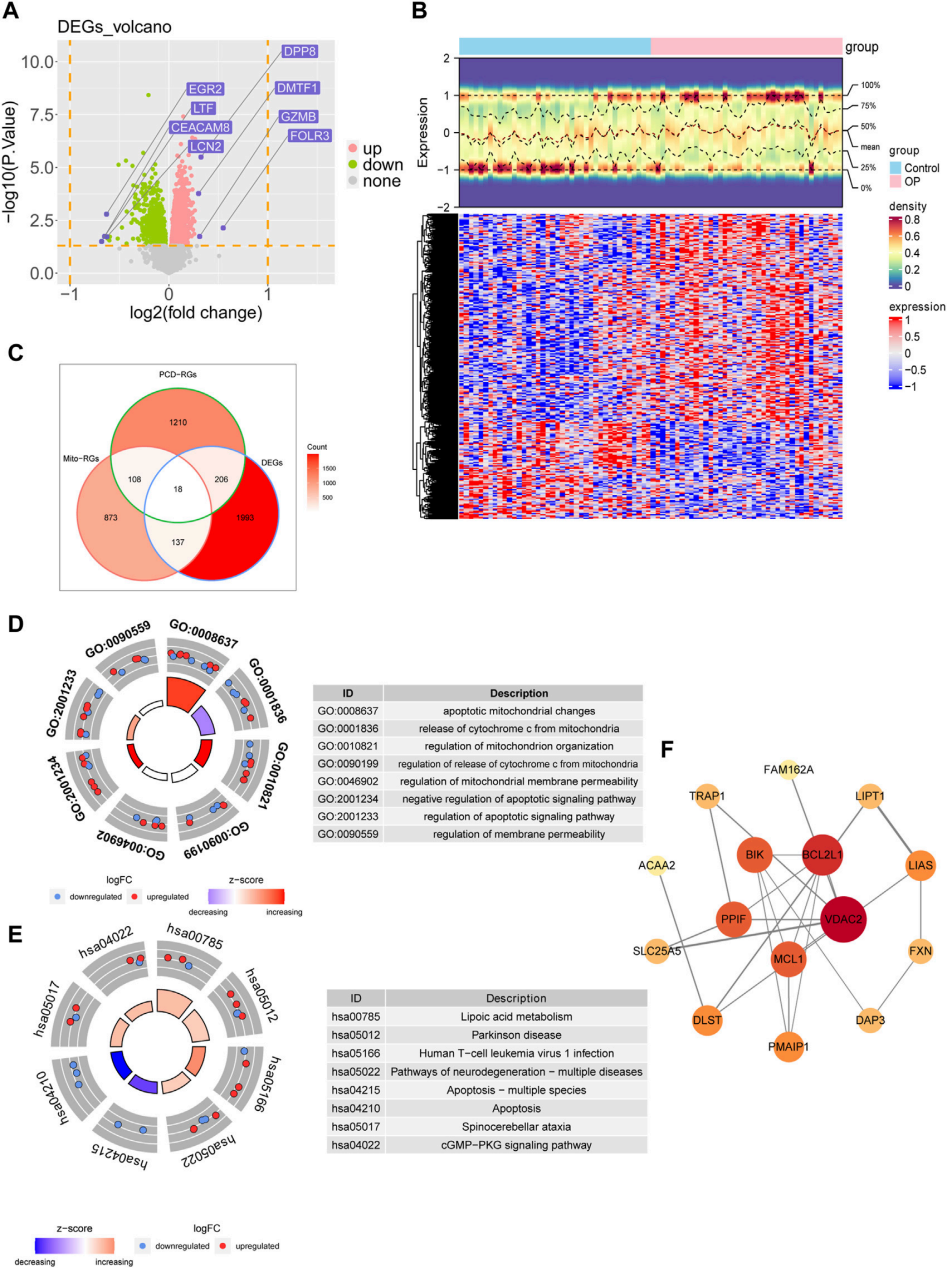
Preliminary identification and analysis of candidate genes. **(A)** Differential volcano plot analysis of the GSE56815 dataset. **(B)** Differential heat map analysis of the GSE56815 dataset. **(C)** Analysis of differential genes with Mito-RGs and PCD-RGs **(D)** GO enrichment analysis. **(E)** KEGG enrichment analysis. **(F)** Reconstruction of the PPI network.

Based on differential expression analysis, 2,354 DEGs were identified between the OP and control groups. Of these, 1,597 DEGs were upregulated in OP, whereas 757 were downregulated (Figures 1A, B). Based on the intersection of 2,354 DEGs, 1,136 MRGs, and 1,548 PCD-RGs, 18 candidate genes were identified (Figure 1C). Enrichment analyses were performed for the candidate genes to preliminarily explore the biological functions in which they were implicated. The results demonstrated significant enrichment of these candidate genes in 95 GO entries, comprising 74 BPs, 17 CCs, and 4 MFs. In addition, these genes were associated with 17 KEGG pathways. Specifically, the enriched GO entries encompassed crucial biological functions such as “regulation of apoptotic signaling pathway,” “regulation of mitochondrion organization,” and “negative regulation of the apoptotic signaling pathway” (Figure 1D). Furthermore, the enriched KEGG pathways included “pathways of neurodegeneration-multiple diseases,” “apoptosis,” “cGMP-PKG signaling pathway,” and others (Figure 1E). A PPI network was constructed based on the candidate genes containing 15 nodes and 22 edges. In particular, there were more interactions between VDAC2, BCL2L1, and MCL1 and other genes (Figure 1F).

### 3.2 Genes encoding death-associated protein 3 (DAP3), Bcl-2-interacting killer (BIK), and acetyl-CoA-acyltransferase 2 (ACAA2) were identified as biomarkers

**FIGURE 2:**
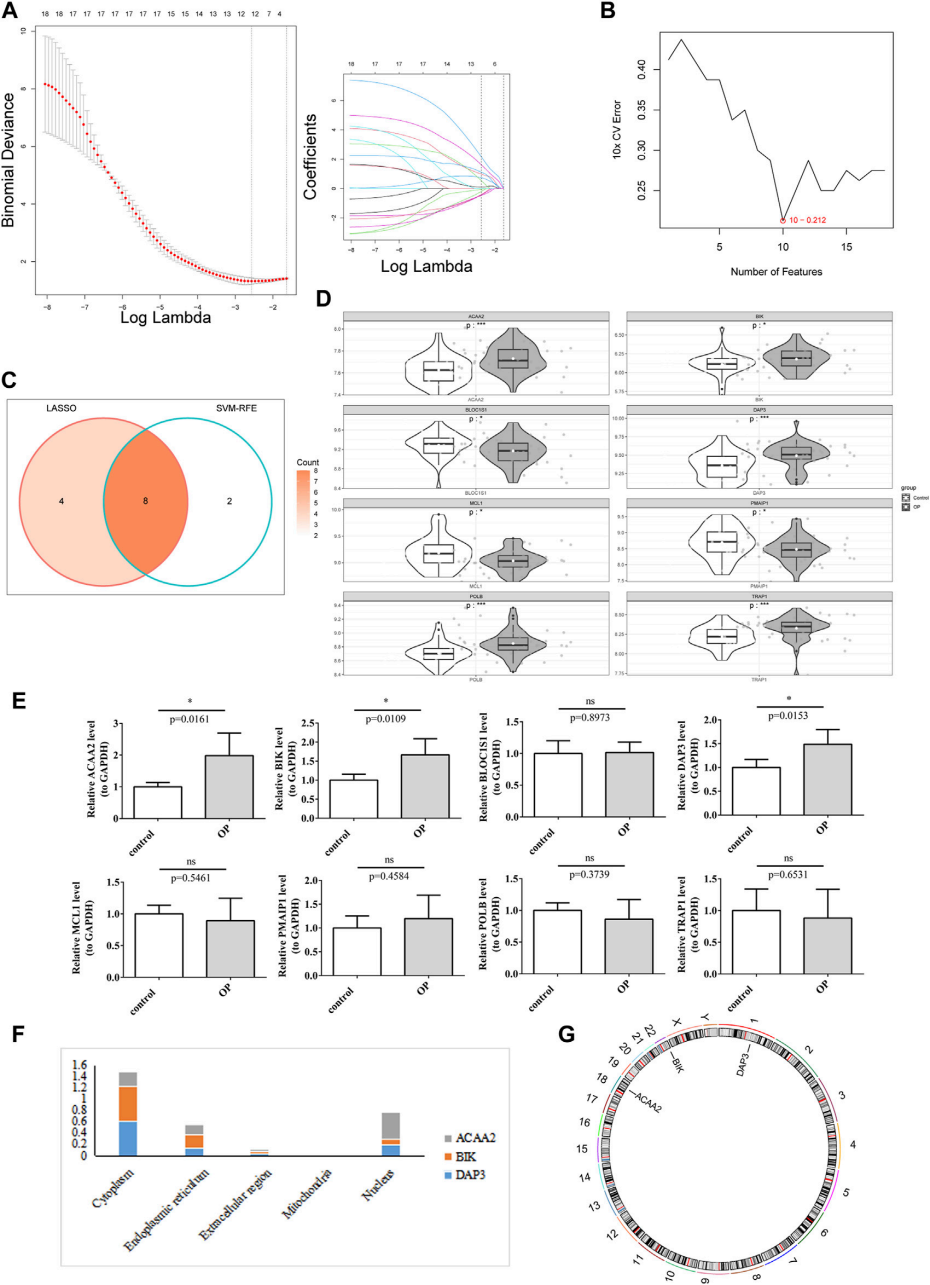
*DAP3*, *BIK*, and *ACAA2* were identified as biomarkers. **(A)** LASSO regression analysis. **(B)** SVM-RFE algorithm regression analysis. **(C)** Convergence of the LASSO and SVM-RFE algorithms. **(D)** Expression of key genes in the training data set. White represents disease samples, gray portrays normal samples. The statistical method used is the Wilcox test. “*” indicates *p* < 0.05, “**” symbolizes *p* < 0.01, “***” signifies *p* < 0.001, “****” depicts *p* < 0.0001, and “ns” denotes no significant difference. **(E)** Expression of key genes in qPCR. The expression of DAP3, BIK, and ACAA2 exhibited significant disparities (*p* < 0.05). **(F)** Subcellular localization prediction of biomarkers. **(G)** Chromosomal localization of biomarkers.

Screening of candidate genes by LASSO regression analysis (Figure 2A) and the SVM-RFE algorithm (Figure 2B) yielded 12 and 10 characterized genes, respectively. By crossing these two parts of the characterized genes, eight candidate biomarkers were identified: *DAP3*, *POLB*, *BLOC1S1*, *MCL1*, *BIK*, *PMAIP1*, *TRAP1*, and *ACAA2* (Figure 2C). The Wilcoxon test showed that in the OP group, *ACAA2*, *BIK*, *DAP3*, *POLB*, and *TRAP1* were markedly upregulated, whereas *BLOC1S1*, *MCL1*, and *PMAIP1* were visibly downregulated compared to the control group (Figure 2D). The qRT-PCR analysis indicated that *DAP3*, *BIK*, and *ACAA2* exhibited expression patterns consistent with the dataset and were significantly elevated in the OP group (Figure 2E). Consequently, *DAP3*, *BIK*, and *ACAA2* were identified as potential biomarkers. Subcellular and chromosomal localization analyses were performed to investigate the distribution of biomarkers. The results demonstrated that BIK and DAP3 were mainly expressed in the cytoplasm, whereas ACAA2 was mainly localized in the nucleus (Figure 2F). *BIK*, *DAP3*, and *ACAA2* are located on chromosomes 22, 1, and 18, respectively (Figure 2G).

### 3.3 The nomogram displayed remarkable potency in prognosticating OP

**FIGURE 3:**
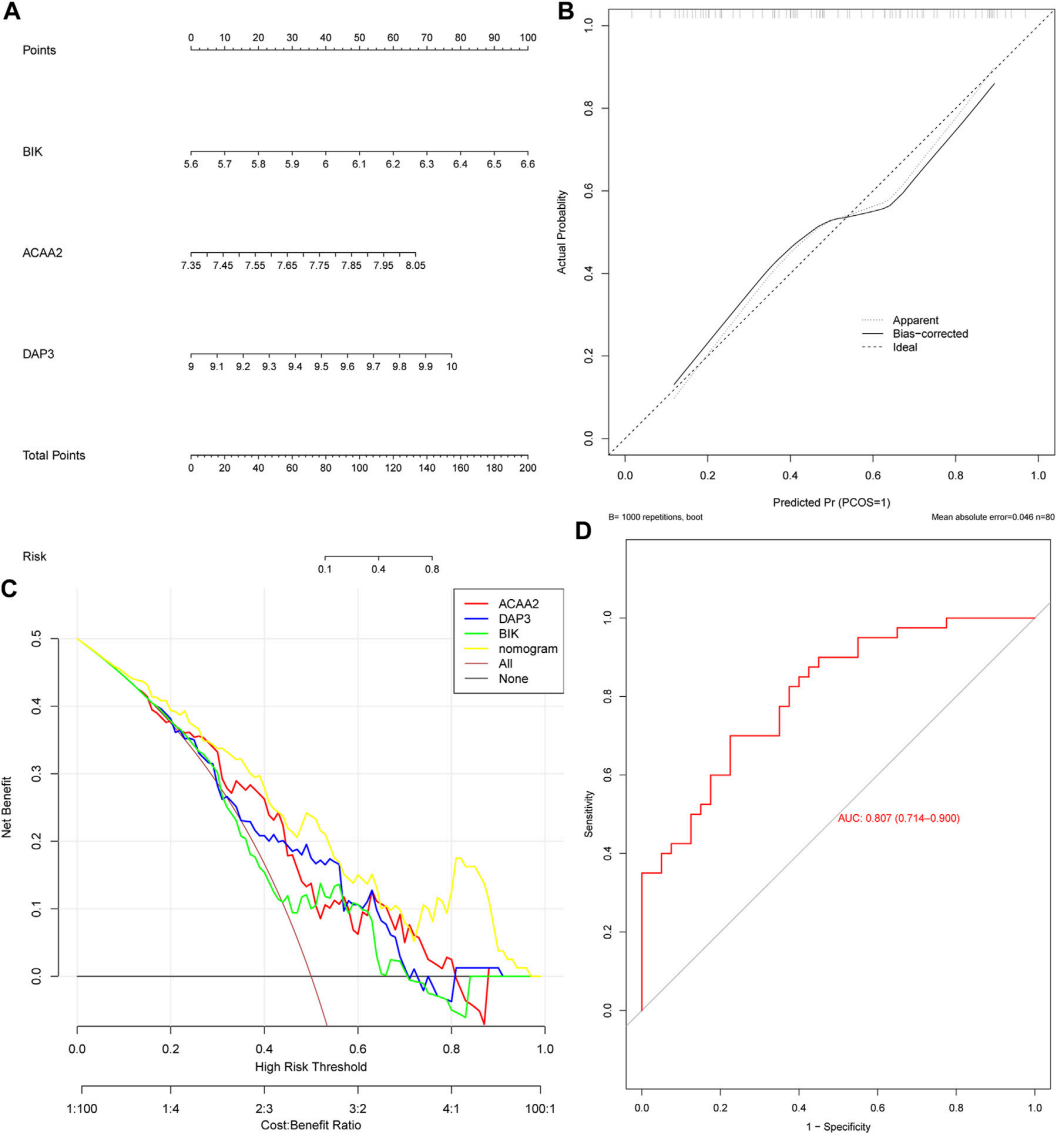
The nomogram exhibited excellent predictive potential for OP. **(A)** Nomogram construction. Evaluation of the diagnostic model of nomogram: **(B)** Calibration curve and **(C)** Decision curve. **(D)** ROC curve.

Based on the identified biomarkers *DAP3*, *BIK*, and *ACAA2*, a nomogram was constructed to assess the ability of the biomarkers to predict OP (Figure 3A). We also evaluated its predictive ability. Specifically, the calibration curve demonstrated a close-to-unity slope for the nomogram (Figure 3B), whereas decision curve analysis indicated that patients with OP could benefit from the constructed diagnostic model (Figure 3C). Additionally, the ROC curve exhibited an area under the curve value of 0.807 (Figure 3D). Collectively, these findings substantiate that the predictive ability of the nomogram constructed based on biomarkers for OP exhibited a certain degree of accuracy.

### 3.4 Biomarkers were linked to oxidative phosphorylation

**FIGURE 4:**
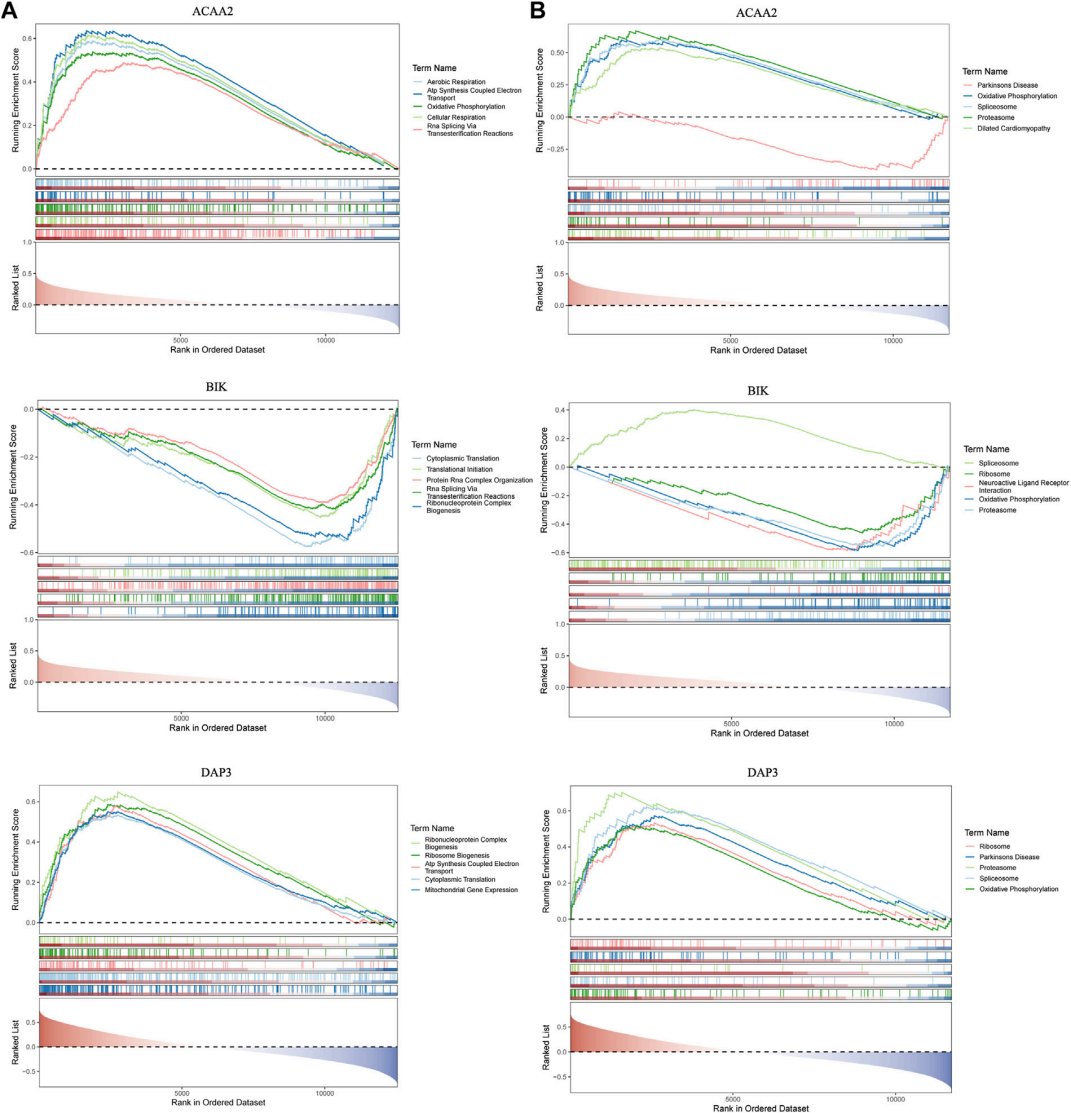
GSEA enrichment analysis. GSEA enrichment analysis based on **(A)** KEGG and **(B)** GO.

GSEA was used to probe the signaling pathways involving *DAP3*, *BIK*, and *ACAA2*. Based on the GO functional enrichment gene set, *DAP3* and *BIK* were co-enriched for “Cytoplasmic Translation” (Figure 4A). Based on the KEGG functional enrichment gene set, *DAP3*, *BIK*, and *ACAA2* were co-enriched in “oxidative phosphorylation,” “proteasome,” and “spliceosome” (Figure 4B).

### 3.5 Central memory CD4^+^ T cells exhibited substantial negative correlation with *DAP3*

**FIGURE 5:**
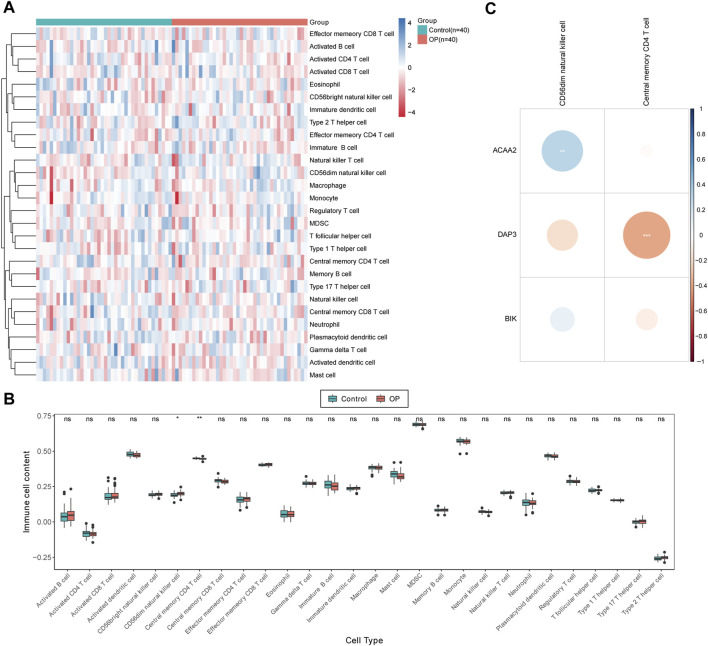
Immunoinfiltration analysis. **(A)** Immune score heat map of immune cell types in different samples obtained by using ssGSEA algorithm. **(B)** Box plot of immune scores acquired by using ssGSEA algorithm for immune cell types in different samples between OP patients and normal controls. The statistical method was Wilcox.test; ns indicates no significant difference, “*” symbolizes *p* < 0.05, “**” depicts *p* < 0.01. **(C)** Differential immune cell and biomarker correlation heat map. Correlation analysis revealed a highly significant positive correlation between CD56dim natural killer cells and ACAA2 (cor = 0.291, *p* = 0.009), while a highly significant negative correlation was observed between central memory CD4^+^ T cells and DAP3 (cor = −0.381, *p* = 0.0004).

The distribution of the 28 immune cell infiltration scores between the OP and control groups in the GSE56815 dataset has been illustrated in Figure 5A. Significant differences were observed between the control and OP groups in terms of CD56dim natural killer cells and central memory CD4^+^ T cells at a significance level of *p* < 0.05. Specifically, compared to the control group, the infiltration scores of CD56dim natural killer cells were significantly higher in the OP group, whereas central memory CD4^+^ T cells exhibited an inverse relationship (Figure 5B). Furthermore, correlation analysis revealed a highly significant positive correlation between CD56dim natural killer cells and *ACAA2* (cor = 0.291, *p* = 0.009), whereas a highly significant negative correlation was observed between central memory CD4^+^ T cells and DAP3 (cor = −0.381, *p* = 0.0004; Figure 5C; Supplementary Table S3).

### 3.6 *DAP3*, *BIK*, and *ACAA2* were regulated by multiple factors

**FIGURE 6:**
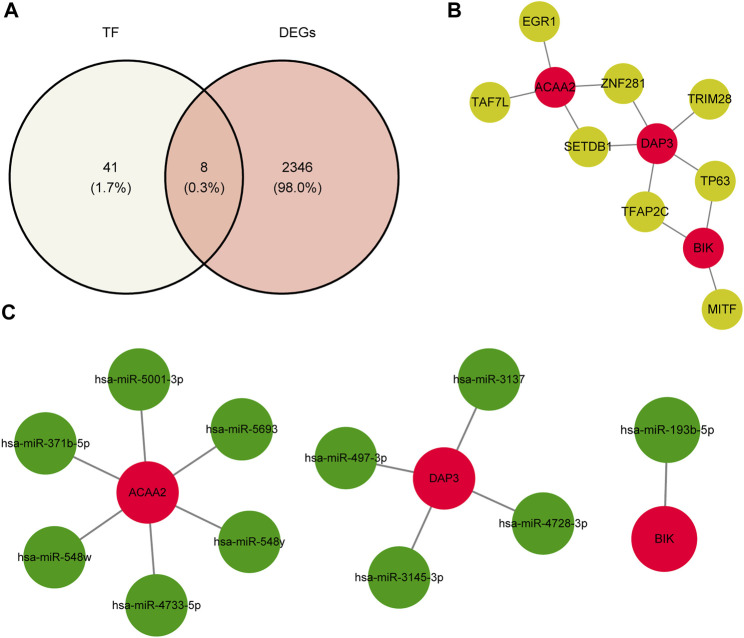
TF-biomarker regulatory network construction. **(A)** Relevant acquisition of TFs. **(B)** Construction of TF-biomarker network map. **(C)** Construction of miRNA-biomarker network map.

Using the NetworkAnalyst database, 24, 15, and 27 TFs were predicted to regulate the expression of DAP3, BIK, and ACAA2. After deduplication and merging, 49 TFs were identified. Eight key TFs were identified by overlapping 49 TFs and 2,354 DEGs (Figure 6A). Based on the key TFs and biomarkers identified, an mRNA-TF network containing 11 nodes and 12 edges was constructed. In this regulatory network, SETDB1 and ZNF281 regulate both *ACAA2* and *DAP3*, whereas TP63 and TFAP2C regulate both *DAP3* and *BIK* (Figure 6B). Additionally, 11 key miRNAs were identified by analyzing the intersection of the predicted miRNAs from the two databases. Specifically, there were one, four, and six miRNAs targeting *BIK*, *DAP3*, and *ACAA2*, respectively (Figure 6C). *ACAA2* is regulated by several miRNAs, including hsa-miR-371b-5P, hsa-miR-548w, and hsa-miR-4733-5p.

### 3.7 A stable binding force was observed between the drugs and biomarkers

**FIGURE 7:**
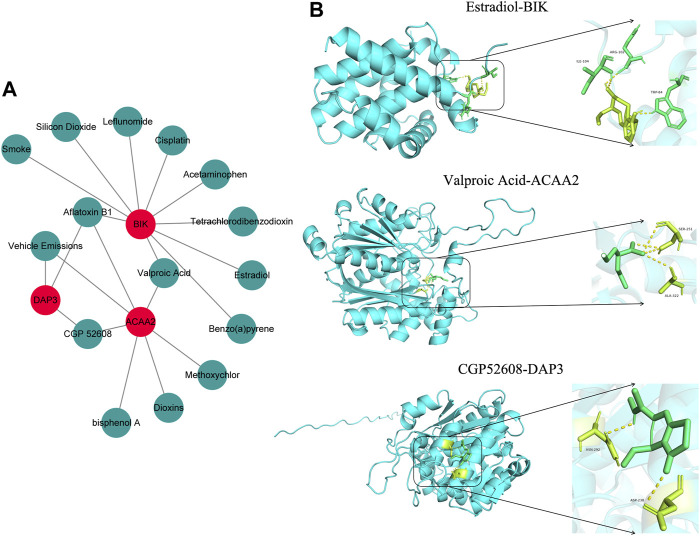
Drug prediction and molecular docking. **(A)** Drug-biomarker network map. **(B)** Visualization of drug molecular docking results.

Based on the screening criteria of reference count ≥1, we predicted 3, 10, and 7 drugs targeting *DAP3*, *BIK*, and *ACAA2*, respectively. Subsequently, a drug-biomarker network containing 18 nodes and 20 edges was constructed. Aflatoxin B1 simultaneously targeted *DAP3*, *BIK*, and *ACAA2* (Figure 7A; Supplementary Table S4). Based on the reference count, estradiol, valproic acid, and CGP52608 were selected for molecular docking analysis using *BIK*, *ACAA2*, and *DAP3*. The results revealed the formation of covalent bonds between BIK and amino acid residues ARG-102, ILE-04, and TRP-84 of estradiol (binding energy = −5.9 kcal/mol). ACAA2 was found to form covalent bonds with amino acid residues SER-251 and ALA-322 of valproic acid (binding energy = −5.0 kcal/mol), while DAP3 formed covalent bonds with amino acid residues ASN-292 and ASP-238 of CGP52608 (binding energy = −5.2 kcal/mol; Figure 7B; Table 1).

**TABLE 1:** Docking molecules and genes.

Chemical name	Gene symbol	PDBID	Reference count	Affinity (kcal/mol)	Residue
Estradiol	*BIK*	7QTW	Bedoui et al. (2020)	−5.9	ARG-102, ILE-04, TRP-84
Valproic acid	*ACAA2*	predicted	Walker and Shane (2023)	−5.0	SER-251, ALA-322
CGP52608	*DAP3*	predicted	Walker and Shane (2023)	−5.2	ASN-292, ASP-238

## 4 Discussion

OP is the primary cause of morbidity and mortality among the elderly worldwide. Several studies have shown that apoptosis is a key factor in OP (Yu and Wang, 2000; Zhang et al., 2019). Mitochondria play a vital role in preserving cellular functions, such as redox balance, calcium equilibrium, energy generation, metabolic processes, and cell demise (Spinelli and Haigis, 2018; Ventura-Clapier et al., 2019). Studies have suggested that irregularities in mitochondrial mass, marked by abnormal oxidative stress, dynamics, and autophagy, have a significant impact on the management of apoptosis linked to bone metabolism (Indo et al., 2017; Cai et al., 2019; Jin et al., 2020; Xu et al., 2021; Li W. et al., 2023). This implies that targeting mitochondrial function and apoptosis may be crucial therapeutic strategies for the treatment of OP.

### 4.1 Enrichment pathways of candidate genes

This study identified 18 candidate genes that are differentially expressed and involved in mitochondria and PCD. The functions of these genes were analyzed through KEGG and GO enrichment pathways, including apoptosis-related pathways, mitochondrial function-related pathways, and the cGMP-PKG signaling pathway. Apoptosis, a type of PCD, is a controlled cellular demise process triggered by the organism to maintain balance and includes death receptors such as TNFR and Fas in the extrinsic pathway of apoptosis and the intrinsically regulated apoptotic pathway mediated by mitochondria (Maiuri et al., 2007; Fischer and Haffner-Luntzer, 2022). Signaling pathways like PI3K/Akt, ERK5, JNK, Wnt/β-catenin, NF-κB, and P38 are involved in mitochondrial pathway apoptosis during OP formation, regulating caspase, Bcl-2 family proteins, and other key targets to affect OB apoptosis (Liu et al., 2024). Studies have shown that the PI3K pathway promotes OB proliferation and differentiation while inhibiting apoptosis in the mitochondrial pathway, ultimately promoting bone formation and increasing bone mass at the tissue level (Meng et al., 2023). JNK is not only involved in inducing apoptosis through the endoplasmic reticulum pathway but also plays a crucial role in inducing apoptosis through the mitochondrial pathway (Guo et al., 2016). The inhibition of the JNK pathway can effectively suppress OB apoptosis, which is beneficial for the treatment of OP. Recent studies have indicated that targeting the NO-cGMP-PKG pathway is pivotal in regulating bone homeostasis and has the potential as an approach for OP treatment (Pal et al., 2020; Friebe et al., 2023). Although nitrates initially garnered attention due to their side effects, soluble guanosine receptor agonists are now FDA-approved for the treatment of pulmonary hypertension and erectile dysfunction (Kim et al., 2021).

### 4.2 Functional analysis of biomarkers

Our OP-related datasets were acquired from the GEO database, whereas the literature was referenced to obtain PCD-RGs and mito-RGs. Bioinformatic analysis and machine learning techniques were used to identify the OP biomarkers *ACAA2*, *DAP3*, and *BIK*. *ACAA2* assumes a pivotal role in fatty acid oxidation. The enzyme encoded by *ACAA2* catalyzes the final step of the fatty acid β-oxidation pathway, contributing to the elongation and degradation of mitochondrial fatty acids. Acetyl-CoA, the final result of lipid β-oxidation, is also important in the creation of ketone bodies. *ACAA2* contains an unremovable amino-terminal signal for targeting and is associated with a range of biological functions. For instance, it influences preadipocyte differentiation in sheep, affecting body fat deposition and the lean meat rate (Han et al., 2021). Excessive accumulation of body fat is associated with obesity, diabetes, fatty liver disease, gallbladder disease, hypertension, and endocrine disorders. Although the direct association between *ACAA2* and bone metabolism remains unclear, its role in fatty acid oxidation suggests potential connections with metabolic diseases, such as obesity and diabetes. These conditions may indirectly influence bone metabolism (Villuendas et al., 2006). Research indicates that ACAA2 expression levels are correlated with the rate of glycolysis and are significant in type 2 diabetes progression (Zhao et al., 2023). DAP3 is a serine/threonine protein kinase known for its pro-apoptotic activity and is primarily associated with cell death. *In vitro* experiments have demonstrated its capability to induce apoptosis, steer recipient cells toward programmed death, and impede cell growth and proliferation (Wazir et al., 2015). Moreover, the cell cycle can be controlled by DAP3 through the inhibition of cell proliferation-associated genes such as cyclin D1 and CDK2, which in turn inhibits the progression of the normal cell cycle and result in cell cycle arrest at either the G0/G1 or G2/M phase, ultimately suppressing abnormal cell growth (Song et al., 2023). However, direct evidence linking *DAP3* to bone metabolism is lacking. Bone metabolism is a multifaceted biological process that involves various cells, hormones, and molecules. Although some proteins related to apoptosis and cell cycle control may indirectly affect bone metabolism, extensive investigation is required to clarify the precise function of *DAP3* in this mechanism. *BIK* constitutes part of the BH3-only protein group of the BCL2 homology domain 3 and assumes a role in the mitochondrial apoptotic pathway (Kale et al., 2018). The protein encoded by *BIK* affects cell viability or apoptosis by interacting with other members of the BCL2 family (Hatok and Racay, 2016). A burgeoning number of studies have confirmed the significance of apoptosis within the mitochondrial pathway in the regulation of bone metabolism (Fortner et al., 2017; Wang et al., 2018; Gao et al., 2022). Therefore, we postulate that targeting consistent apoptosis through enhancement of mitochondrial status represents a pivotal therapeutic strategy for addressing osteoporosis.

### 4.3 GSEA enrichment analysis and mechanism of action

GSEA enrichment analysis revealed that the three common genes were closely associated with oxidative phosphorylation pathway signaling. The cellular energy metabolism pathways consists of glycolysis, which is dominated by anaerobic respiration, and oxidative phosphorylation, which is dominated by aerobic respiration. The oxidative phosphorylation pathway is particularly relevant to osteogenic and osteoclastic differentiation. Shares et al. (2018) observed a significant increase in endogenous ATP and oxygen consumption during osteogenic differentiation of MSCs, indicating a reliance on mitochondrial oxidative phosphorylation for energy. Snoeck (2017) also found that the energy for osteogenic and OB differentiation of MSCs is mainly provided through the oxidative phosphorylation pathway. Furthermore, Kim et al., 2021; Ledesma-Colunga et al., 2023 demonstrated that inhibition of the mitochondrial oxidative phosphorylation energy supply pathway hindered osteoclast differentiation, underscoring the crucial role of this pathway in osteoclast function. In conclusion, targeting the oxidative fatty acid metabolic pathway is imperative for enhancing energy supply during the osteoblastic process, thereby offering a promising therapeutic approach for managing osteoporosis.

### 4.4 Immune infiltration and drug prediction analysis

The bone marrow contains hematopoietic stem cells and various mature immune cells that influence OBs and osteoclasts, which are crucial for bone structural integrity and repair. This interaction between cells leads to bone remodeling, a dynamic process of formation and resorption (Brylka and Schinke, 2019; Saxena et al., 2021). Our research uncovered noteworthy differences in CD56dim natural killer cells and central memory CD4^+^ T cells between the control and OP cohorts. Natural killer cells are part of the innate immune system and are associated with cellular senescence, whereas CD4^+^ T cells contribute to bone loss through the osteoimmune system (Antonangeli et al., 2019). The discovery that activated CD4^+^ T cells regulate osteoclast bone resorption is one of the driving forces for the development of bone immunology (Okamoto and Takayanagi, 2023). Through further elucidation of the interplay between bone and the immune system, insights into the intricate relationship between osteogenesis and inflammation in the realm of bone immunology are unveiled. Consequently, as investigations into helper T cell subsets and rare lymphoid cells progress within the field of immunology, it is increasingly evident that diverse types of T cells exert multifaceted effects on bone metabolism contingent upon the immune milieu. Comprehending the distinct impacts of T cells on bone is pivotal for unraveling bone immunomodulatory networks across various biological contexts.

The drug prediction results suggested stable binding between the drugs and biomarkers, with estrogen demonstrating a protective effect on human bone OBs by exhibiting an anti-apoptotic effect. Estrogen inhibits OB apoptosis and promotes bone formation (Lu and Tian, 2023). Additionally, studies have shown that valproate prevents glucocorticoid-induced femoral head necrosis in rats (Zhou et al., 2018). Additionally, prior research has verified the anti-osteoporotic potential of most drug predictions, indicating the clinical significance of predicted biomarkers.

### 4.5 Limitations and prospects

There are certain limitations associated with this study. The relatively small number of clinical samples may introduce bias into the outcomes. Additionally, there is a lack of support for further validation through animal experiments. To address these deficiencies, we intend to conduct animal studies to enhance the reliability of the biomarkers and will continue monitoring the progress of ACAA2, DAP3, and BIK in relation to osteoporosis.

## 5 Conclusion

This study used bioinformatics and qRT-PCR analyses to identify three biomarkers associated with PCD and mitochondria in OP, namely, *ACAA2*, *DAP3*, and *BIK*. Enrichment and immune infiltration analyses, regulatory network construction, drug prediction, and additional assessments were performed to investigate the potential mechanisms of action of these biomarkers.

## Data Availability

The codes for analysis and data are available online at https://github.com/yangxiu1992/. The names of the repository/repositories and accession number(s) can be found in the article/Supplementary Material.

## Abbreviations

BMD, bone mineral density; BMSCs, bone marrow mesenchymal stem cells; BP, biological processes; CC, cellular components; DAP3, death-associated protein 3; DEGs, differentially expressed genes; GEO, Gene Expression Omnibus; GO, Gene Ontology; GSEA, gene set enrichment analysis; KEGG, Kyoto Encyclopedia of Genes and Genomes; MF, molecular functions; miRNAs, microRNAs; MRGs, mortality factor-related genes; OB, osteoblast; OP, osteoporosis; PCD, programmed cell death; PCD-RGs, genes linked to programmed cell death; PDB, Protein Data Bank; PPI, protein-protein interaction; qRT-PCR, real-time polymerase chain reaction; ROS, reactive oxygen species; SVM-RFE, support vector machine recursive feature elimination; TFs, transcription factors.
