# Supplementary material for: Identification and experimental validation of programmed cell death- and mitochondria-associated biomarkers in osteoporosis and immune microenvironment
Source: Front Genet. 2024 Jul 26;15:1439171. doi: 10.3389/fgene.2024.1439171 (PMC11310001; doi:10.3389/fgene.2024.1439171)
Supplement: Supplementary file 3 [file Table1.DOCX]

Supplementary Material

# Supplementary Tables

**Supplementary Table 1.** Detailed list of the 1,548 genes related to programmed cell death

(Table uploaded in a separate excel file)

**Supplementary Table 2.** PCR primer sequences

| Primer | Sequence | |
| --- | --- | --- |
| DAP3 F | GGCATAACACGGGTGAGGAA | |
| DAP3 R | TAATTCCTCGGGGGCAATCG | |
| POLB F | TGGAAAAGATTCGGCAGGATG |  |
| POLB R | CAGATGGACCAATGCCACTAAC |  |
| BLOC1S1 F | CTGTCCCGCCTCCTAAAAGA |  |
| BLOC1S1 R | GATAGCCTCTCGCCTCCTCT |  |
| MCL1 F | CCTTCCAAGGATGGGTTTGTG |  |
| MCL1 R | TGCCAAACCAGCTCCTACTC |  |
| BIK F | CCTGGGTCTGGCTTTCATCT |  |
| BIK R | AGTGTGGTGAAACCGTCCAT |  |
| PMAIP1 F | GGGCCACGAGGAACAAGTAG |  |
| PMAIP1 R | GAAACGTGCACCTCCTGAGA |  |
| TRAP1 F | GCTCTGGGAGTACGACATGG |  |
| TRAP1 R | TAATCGAGTGCAGGGGTTCC |  |
| ACAA2 F | AGACAATGCAGGTAGACGAGC |  |
| ACAA2 R | ACCCATGATAGAGGGATCACATC |  |
| Internal reference-GAPDH F | CGAAGGTGGAGTCAACGGATTT |  |
| Internal reference-GAPDH R | ATGGGTGGAATCATATTGGAAC |  |

**Supplementary Table 3.** Immune cell infiltration scores

|  | CD56dim natural killer cell | Central memory CD4 T cell |
| --- | --- | --- |
| *ACAA2* | 0.008894201 | 0.8300396 |
| *DAP3* | 0.143795529 | 0.000491058 |
| *BIK* | 0.364350775 | 0.477565897 |

**Supplementary Table 4.** Drug-biomarker network

(Table uploaded in a separate excel file)
